# Toll-like receptor 3 activation promotes joint degeneration in osteoarthritis

**DOI:** 10.1038/s41419-022-04680-5

**Published:** 2022-03-11

**Authors:** Josef Stolberg-Stolberg, Annika Boettcher, Meike Sambale, Sina Stuecker, Joanna Sherwood, Michael Raschke, Thomas Pap, Jessica Bertrand

**Affiliations:** 1 Institute of Musculoskeletal Medicine, University Hospital Muenster, Albert-Schweitzer-Campus 1, Building D 3, 48149 Muenster, Germany; 2 Department of Trauma-, Hand- and Reconstructive Surgery, University Hospital Muenster, Albert-Schweitzer-Campus 1, Building W 1, 48149 Muenster, Germany; 3 Department of Orthopaedic Surgery, Otto-von-Guericke University, Leipziger Straße 44, 39120 Magdeburg, Germany

**Keywords:** Experimental models of disease, Trauma

## Abstract

Osteoarthritis (OA) is characterized by cartilage degradation that is induced by inflammation. Sterile inflammation can be caused by damage-associated molecular patterns that are released by chondrocytes and activate pattern recognition receptors. We evaluate the role of toll-like receptor-3-activating RNA in the pathogenesis of OA. Toll-like receptor 3 (TLR3) was detected by semiquantitative reverse transcriptase PCR, western blotting and microscopy. Rhodamine-labelled poly(I:C) was used to image uptake in chondrocytes and full-thickness cartilage. The production of IFNβ in chondrocytes after stimulation with poly(I:C) as well as in the synovial fluid of OA patients was measured using ELISA. Chondrocyte apoptosis was chemically induced using staurosporine. Immunohistochemistry was performed to examine TLR3 expression and apoptosis in human and murine OA cartilage. RNA in synovial fluid was quantified by RiboGreen assay. Destabilisation of the medial meniscus was performed in TLR3^−/−^ and wildtype mice. OA was assessed after eight weeks using OARSI score. TLR3 expression was confirmed by western blot and RT-PCR. Poly(I:C) was internalised by chondrocytes as well as cartilage and caused an increase of IFNβ production in murine (11.46 ± 11.63 (wo) to 108.7 ± 25.53 pg/ml; *N* = 6) and human chondrocytes (1.88 ± 0.32 (wo) to 737.6 ± 130.5 pg/ml; *N* = 3; *p* < 0.001). OA cartilage showed significantly more TLR3-positive (KL0 = 0.22 ± 0.24; KL4 = 6.02 ± 6.75; *N* ≥ 15) and apoptotic chondrocytes (KL0 = 0.6 ± 1.02; KL4 = 9.78 ± 7.79; *N* ≥ 12) than healthy cartilage (*p* < 0.001). Staurosporine-induced chondrocyte apoptosis causes a dose-dependent RNA release (0 ng/ml = 1090 ± 39.1 ng/ml; 1000 ng/ml=2014 ± 160 ng/ml; *N* = 4; p < 0.001). Human OA synovial fluid contained increased concentrations of RNA (KL0-2 = 3408 ± 1129 ng/ml; KL4 = 4870 ± 1612ng/ml; *N* ≥ 7; *p* < 0.05) and IFNβ (KL0-2 = 41.95 ± 92.94 ng/ml; KL3 = 1181 ± 1865ng/ml; *N* ≥ 8; *p* < 0.05). TLR3^−/−^ mice showed reduced cartilage degradation eight weeks after OA induction (OARSI WT = 5.5 ± 0.04; TLR3^−/−^ = 3.75 ± 1.04; *N* ≥ 6) which was accompanied by gradually decreasing levels of TUNEL-positive cells (WT = 34.87 ± 24.10; TLR3^−/^ = 19.64 ± 7.89) resulting in decreased IFNβ expression (WT = 12.57 ± 5.43; TLR3^−/−^ = 6.09 ± 2.07) in cartilage (*p* < 0.05). The release of RNA by apoptotic chondrocytes thus activating TLR3 signalling is one possible way of perpetuating inflammatory cartilage changes. The inhibition of TLR3 could be a possible therapeutic target for OA treatment.

## Introduction

Osteoarthritis (OA) is a progressive joint disease, which is associated with severe pain and impairment of movement which results in a significant reduction of the quality of life. OA is a degenerative disease that is characterized by progressive structural changes in joint tissues. Especially the articular cartilage is associated with cartilage fibrillation and erosions accompanied by chondrocyte hypertrophic differentiation and changes in extracellular matrix composition. Different factors, including cytokines, growth factors and Wnts have been identified as being involved in OA disease progression. Innate inflammatory signals such as damage-associated molecular patterns (DAMPs) have been described to activate pattern-recognition receptors (PRRs), which induce the expression of matrix metalloproteinase (MMP) as well as disintegrin and metalloprotease with thrombospondin motif (ADAMTS), resulting in cartilage thinning with progressive loss of proteoglycans and collagen [1, 2].

The expression of different TLRs (toll-like receptors) in cartilage has been described [3]. Current research, however, focuses mainly on the role of TLR2 and 4 in OA [4, 5]. These TLRs have been studied in cartilage and chondrocytes extensively, however, not much is known about the role of nucleic-acid binding TLRs such as TLR3 and TLR7-9.

Toll-like receptor 3 (TLR3) is a PRR and type I transmembrane receptor with an extracellular domain located in endosomes [6, 7]. Upon RNA or poly(I:C) binding and receptor dimerization TRIF is recruited inducing an activation pathway of IRF3, NFkB, INFβ and proinflammatory cytokine gene expression [8, 9]. The group of TLRs plays an essential role in the pathogenesis of chronic inflammatory disease such as rheumatoid arthritis, interacting with endogenous ligands that originate from degraded extracellular matrix or dying cells [10, 11]. Furthermore, epidemiologic data shows that polymorphisms in the promoter region of TLR3 are associated with primary osteoarthritis (OA) [12]. Gene expression studies identified a range of PRRs in OA cartilage including TLR3, retinoic acid–inducible gene 1 and nucleotide-binding oligomerization domain–like receptor X1, which are capable of binding endogenous nucleic acids [13]. Because MMP expression in chondrocytes is up-regulated after Poly(I:C) stimulation, the modulation of TLR signalling was initially suggested as a potential therapeutic strategy [14]. However, the simulation of a viral joint infection by intra-articular poly(I:C) injections induced signs of arthritis that were also seen in TLR3-deficient (TLR3^−/^) mice [15]. Although a recent study suggests that poly(I:C)-induced arthritis is regulated by the TLR3-p38 MAPK-NF-κB Il-33 pathway, which is modulated by the p65 and peroxisome proliferator-activated receptor-γ (PPARγ) complex, the role of TLR3 activation in the pathogenesis of osteoarthritis remains elusive [16].

Current research shows that nucleotides, being a potential ligand for nucleotide binding TLRs, are released during chondrocyte cell death after joint trauma [17]. Furthermore, in vivo chondrocyte depletion models suggest that the cartilage is protected from degenerative changes [18, 19]. Thus, endogenous ligands might contribute to degenerative pathways in OA. The aim of this study is to investigate whether endogenous RNA could activate TLR3 in cartilage, thereby inducing a sterile inflammation and contributing to cartilage degeneration during OA.

## Materials and methods

### OA cartilage samples

Human OA articular cartilage was obtained from patients undergoing joint replacement for knee OA after obtaining written consent (in accordance with the ethical standards of the responsible committee on human experimentation and with the Helsinki Declaration of 1975, as revised in 2000). Healthy cartilage samples were harvested from body donors of the forensic department within 24 h after death (IRRB Magdeburg medical faculty: 23/16). Full thickness cartilage samples were dissected from the main loaded areas of the joint. Safranin-O staining was applied for OARSI scoring [40]. Two independent graders assessed the OARSI score in a blinded manner.

### Chondrocyte culture

Primary human chondrocytes were isolated from OA cartilage samples and cultured with supplemented high glucose Dulbecco’s Modified Eagle’s Medium (DMEM) (PAA Laboratories, Pasching, Germany) at 37 °C and 5% CO_2_ [41]. For nucleotide internalization P1 chondrocytes were analyzed. For all other experiments P2 chondrocytes were used. DMEM was supplemented with 10% fetal calf serum (Biochrom, Berlin, Germany), 1% sodium pyruvat (Thermo Scientific, Waltham, USA) and 1% penicillin/streptomycin/fungizone (PAA Laboratories, Pasching, Germany). Primary murine chondrocytes were isolated from neonatal C57BL/6 J wildtype mice according to Gosset et al. [42].

### Quantitative Reverse-Transcriptase PCR

RNA was isolated from primary human (*N* ≥ 3) chondrocytes or murine chondrocytes (*N* ≥ 3). GenElute™ Mammalian Total RNA Miniprep Kit (Sigma-Aldrich, St. Louis, USA) and DNase I Amplification Grade Kit (Sigma-Aldrich, St. Louis, USA) were used according to the manufacturer’s instructions. First Strand cDNA Synthesis Kit (Life technologies) and oligo(dT) primers were used for transcription. Quantitative RT-PCR was performed using SYBR^®^ Select Master Mix (Sigma-Aldrich, St. Louis, USA). The primers are listed in Table 1. GAPDH was used as housekeeping gene for normalisation. Relative quantification was performed using a standard curve.

### Nucleic Acid Internalization

Human chondrocytes (P1) were plated at a density of 8 × 10^3^ cells/cm^2^ on a glass cover slip in a 24-well plate. Rhodamine-conjugated poly(I:C) (Invivogen, San Diego, USA) or FITC labelled CpG ODN (Invivogen, San Diego, USA) was added and cells were incubated for 24 h and mounted with ROTI^®^ Mount FluorCare DAPI (Carl Roth, Karlsruhe, Germany). Unconjugated rhodamine or FITC (Invivogen, San Diego, USA) at respective concentrations were used as negative control. Murine hip caps were obtained from 4 weeks old mice. Whole cartilage samples were incubated with rhodamine-conjugated poly(I:C), FITC labelled CpG ODN, unconjugated rhodamine or FITC for 36 h. Again, DAPI was used as counterstain. Samples were embedded in TissueTek (Sakura, Alphen aan den Rijn, Netherlands). Each experiment was performed at least 3 times and all samples were analysed using a Zeiss confocal laser scanning system LSM 510 meta.

### IFN-α and -β ELISA

Primary human chondrocytes were seeded in a 24-well plate at a density of 3 × 10^5^ in supplemented DMEM medium and stimulated with 50 µg/ml poly(I:C), CpG-ODN (ODN 2006, Invivogen, San Diego, USA) for 24 h or nucleotides in medium (1% FCS) for 24 h. TLR3 inhibitor CU CPT4a was added (*N* = 4). Additionally, undiluted human synovial fluid of OA patients (*N* > 6) with different KL-scores obtained before knee replacement (after obtaining written consent, in accordance with the ethical standards of the responsible committee on human experimentation and with the Helsinki Declaration of 1975, as revised in 2000) or from the forensic department was analysed (IRRB Magdeburg (blinded for review) medical faculty: 23/16). SF of body donors was obtained within 24 h after death. IFN-α and IFN-β concentrations in the supernatant were measured using the human IFN-α ELISA kit (R&D systems, Wiesbaden, Germany) and IFN-β ELISA kit (Abcam, Cambridge, UK), respectively, according to the manufacturers’ instructions.

### TLR3 immunostaining

C-28/I2 human chondrocytes were seeded at a density of 8 × 10^3^ cells/cm^2^ on a glass cover slip in a 24-well plate (*N* ≥ 3). After 24 h cells were fixed with 4% PFA. Human cartilage sections (*N* ≥ 15) were pre-treated using sodium citrate buffer adjusted to pH6. Anti-TLR3 antibody (1:500, NBP2-24875, Novus, Centennial, USA) or murine IgG diluted 1:50 in Tris-buffered saline (TBS) were applied over night at 4 °C. The Alexa Fluor® 488 anti mouse from donkey was used as secondary antibody. Fluorescence microscopy was performed using Axio Observer.Z1 (Zeiss, Oberkochen, Germany). TLR3 and DAPI positive cells were counted by the help of ImageJ.

### Western blot

Proteins (human and murine *N* ≥ 3) were extracted using RIPA lysis buffer supplemented with Complete Ultra Tablets (Roche) and PhosSTOP (Roche, Grenzach, Germany). TLR3 was detected using 1:1000 diluted anti-TLR3 antibody (ab83338, Abcam, Cambridge, UK) and anti-rabbit-horseradish-peroxidase (3:5000). Anti-GAPDH (#14C10, Cell Signaling, Danvers, USA) diluted 1:1000 was used as loading control.

### Extracellular RNA quantification

The Quanti-iT™ RNA kit (Quanti-iT™ RiboGreen^®^ assay kit, Molecular Probes®, Oregon, USA) was used to according to the manufacturer’s instructions. Synovial fluid (*N* ≥ 7) was diluted in TE buffer (1:5). 100 µL of diluted synovial fluid or supernatant of staurosporine-stimulated primary human chondrocytes ( = 4) were added to a 96-well plate. Quanti-iT™ RNA reagent was diluted 1:200 in TE buffer (10 mM Tris-HCl, EDTA, pH 7.5) and 100 µL was added to each well. Fluorescence intensity (excitation/emission 480/520 nm) was measured after 5 min incubation with a micro-plate reader.

### TLR3 knockdown

Primary human chondrocytes (N = 3) were transformed using jetPRIME® (Illkirch-Graffenstaden, France) and either 50 µM siTLR3 (Silencer® Select, #s8862, AMBION GmbH, Kaufungen, Germany) or 50 µM siScrambled (Silencer® Negative Control #1, AMBION GmbH, Kaufungen, Germany) per well. To measure the knockdown efficacy a semiquantitative RT-PCR was performed.

### DMM-induced experimental OA model

Surgical OA was induced by destabilization of the medial meniscus in littermate male TLR3^−/−^ (N = 8) or wildtype (N = 6) mice (Jackson Laboratory; Stock No.: 005217) at the age of 10 weeks (State Agency for Nature, Environment and Consumer Protection North Rhine-Westphalia, Germany, Project Number 81-02.04.2017.A400) [43]. Sham surgery was done on the contralateral limb by a medial skin incision and wound closure. Animal care was in accordance with institution guidelines. After eight weeks mice were euthanized and both knees were processed for histology. Two independent graders assessed the sections of the tibia and femur in a blinded manner using the OARSI scoring system [44].

### IFN beta staining

Murine cartilage (*N* ≥ 6) sections were pre-treated using trypsin. Anti-IFNβ antibody (NBP1-77288, Novus Biologicals, Centennial, USA) or rabbit IgG diluted 1:400 in Tris-buffered saline (TBS) were applied over night at 4 °C. The Alexa Fluor® 488 anti rabbit from donkey was used as secondary antibody. Fluorescence microscopy was performed using Axio Observer.Z1 (Zeiss, Oberkochen, Germany). IFNβ staining in the cartilage area was quantified using ImageJ.

### TUNEL assay

Murine (*N* ≥ 6) and human (*N* > 8) cartilage sections were stained using the “in situ cell death detection kit” (Merck, Darmstadt, Germany) according to the manufacturer’s instructions. Fluorescence microscopy was performed using Axio Observer.Z1 (Zeiss, Oberkochen, Germany). TUNEL and DAPI positive cells were manually marked and put into relation ((TUNEL positive cells/total number of cells)) *100) with the help of ImageJ.

### Chemically induced apoptosis

Apoptosis in primary human chondrocytes was chemically induced by staurosporine (LKT laboratories, Minnesota, USA) using concentrations of 100 ng/ml, 200 ng/ml, 500 ng/ml and 1000 ng/ml for 24 h [45]. The amount of extracellular RNA in the supernatant was measured using RiboGreen^®^ assay (Thermo Scientific, Waltham, USA).

### Statistical analysis

Data are presented as means + /- SEM (parametric) or median ± 95% CI (non-parametric). According to the data distribution, student’s *t*-test, ordinary one way-ANOVA-analysis, Kruskal-Wallis test or Mann-Whitney-*U-*test were performed using GraphPad Prism Software, V.6.0 h (GraphPad Software Inc), with *p* < 0.05 determining the primary level of significance. All analyses were fully explorative without adjustment for multiple test problems. All results are interpreted accordingly.

## Results

### TLRs are expressed and functional in chondrocytes

**Fig. 1:**
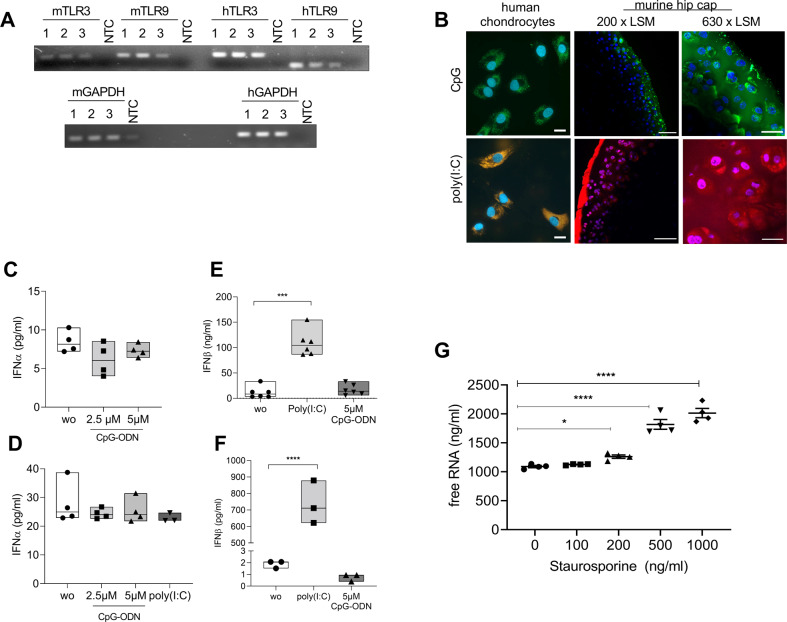
TLRs are expressed and functional in chondrocytes. **A** RT-PCR for TLR3 and 9 expression in primary human and murine chondrocytes. GAPDH served as loading control. **B** Confocal microscopy of labelled CpG (green) and poly(I:C) (red) uptake by either primary human chondrocytes (scale bar 20 µm) or murine hip caps (scale bar 50 µm (centre) 20 µm (right)). DAPI (blue) was used as counterstaining for nuclei. **C** IFNα ELISA of murine chondrocytes stimulated with ODN (one-way ANOVA:F (2, 9) = 2.21, *p* = 0.16, *N* = 4). **D** IFNα ELISA of primary human chondrocytes stimulated with ODN and poly(I:C) (one-way ANOVA:F (3, 11) = 0.75, *p* = 0.55, *N* = 4). **E** IFNβ ELISA of murine chondrocytes stimulated with poly(I:C) or ODN (one-way ANOVA: F (2, 15) = 59.61, *p* < 0.0001, *N* = 6). **F** IFNβ ELISA of primary human chondrocytes stimulated with poly(I:C) or ODN (one-way ANOVA:F (2, 6) = 95.43, *p* = <0.0001, *N* = 3). **G** Staurosporine was used to induce cell death. The release of RNA from human primary chondrocytes after treatment was measured in the culture supernatant using the RiboGreen assay (RM ANOVA: F (4, 12) = 101.2, *p* < 0.0001, *N* = 4). Dunnett’s post-hoc test revealed an increase for 200, 500, and 1000 ng/ml staurosporine treatment compared to the untreated control.

Firstly we evaluated the expression pattern of ds nucleotide-binding TLRs in murine and human chondrocytes. The RT- PCR showed that TLR3 and TLR9 are expressed by human and murine primary chondrocytes (Fig. 1A). To test whether established ligands for TLR3 and TLR9 would be taken up by chondrocytes we incubated monolayer primary human chondrocytes with rhodamine labelled poly(I:C) and FITC labelled CpG-ODN. We observed an intracellular uptake of both labelled nucleotides in vesicular structures of the chondrocytes (Fig. 1B). To understand if the extracellular matrix of cartilage would prevent chondrocytes from nucleotide uptake in vivo, we incubated murine cartilage hip caps with rhodamine labelled poly(I:C) and FITC labelled CpG-ODN. Again, we found the fluorescence for both nucleotide types in intracellular, vesicular structures of the cartilage (Fig. 1B). Both experiments (Fig. 1A, B) were repeated at least three times with different samples. As both established TLR-ligands were taken up by chondrocytes, as well as cartilage, we investigated the functional activation of both TLRs in murine and human primary chondrocytes. The activation of TLR9 using CpG-ODN did not result in an increase in INFα in primary murine (Fig. 1C; wo = 8.45 ± 1.37 pg/ml; 2.5 μM CpG-ODN = 6.17 ± 2.1 pg/ml; 5 μM CpG-ODN = 7.33 ± 0.85 pg/ml; *p* = 0.16, *N* = 4) or primary human chondrocytes (Fig. 1D; wo = 27.89 ± 7.39 pg/ml; 2.5 μM CpG-ODN = 24.36 ± 1.77 pg/ml; 5 μM CpG-ODN = 25.36 ± 4.26 pg/ml; poly(I:C) = 22.91 ± 1.65; *p* = 0.55, *N* = 4). This finding indicates that TLR9 might not be activated in chondrocytes. The stimulation of TLR3 with poly(I:C), however, induced a 10-fold increase in IFNβ expression in murine (Fig. 1E; wo = 11.46 ± 11.63 pg/ml; poly(I:C) = 108.7 ± 25.53 pg/ml; CpG-ODN = 17.04 ± 10.59 pg/ml; *p* < 0.0001, *N* = 6) and more than a 100-fold increase in human chondrocytes (Fig. 1F; wo = 1.88 ± 0.32 pg/ml; poly(I:C) = 737.6 ± 130.5 pg/ml; CpG-ODN = 0.75 ± 0.32 pg/ml; *p* < 0.0001, *N* = 3); indicating that TLR3 might be active in chondrocytes. Next, we used staurosporine to induce chondrocyte death by apoptosis. With increasing staurosporine concentrations we observed a dose-dependent increase of free RNA in the supernatant of the chondrocyte culture showing that chondrocyte apoptosis is associated with TLR3 ligand release (Fig. 1G; 0 ng/ml=1090 ± 39.1 ng/ml (*N* = 4); 100 ng/ml = 1125 ± 10.65 ng/ml (*N* = 4); 200 ng/ml = 1261 ± 57.79 ng/ml; 500 ng/ml = 1818 ± 168 ng/ml; 1000 ng/ml = 2014 ± 160 ng/ml (*N* = 4); 0 ng/ml vs. 200 ng/ml *p* < 0.05; 0 ng/ml vs 500 ng/ml *p* < 0.0001; 0 ng/ml vs 1000 ng/ml *p* < 0.0001).

### TLR3 signalling is activated in OA cartilage

**Fig. 2:**
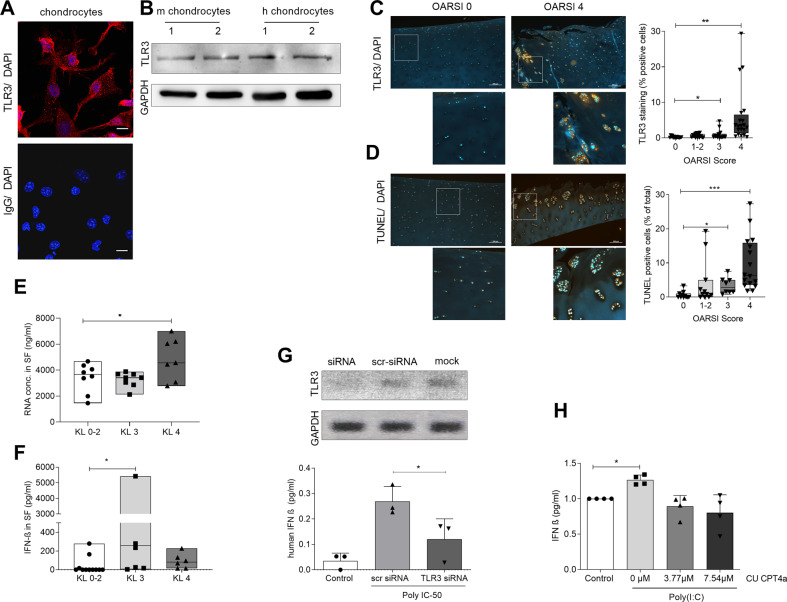
TLR3 signalling is activated in OA cartilage. **A** Confocal imaging TLR3 immunostaining (TLR3: red, DAPI: blue) on C28 chondrocytes. **B** Western Blot for TLR3 in human and murine chondrocytes. GAPDH served as loading control. **C** TLR3 immunostaining in human OA cartilage. The quantification of TLR3 staining revealed an increase of staining with increasing OA grade (Scale bar: 200 µm, Kruskal-Wallis test: F (4, 71) = 34.82, *p* < 0.0001, *N* > 15), (Dunn’s post-hoc, mean rank diff: OARSI 0 vs 1-2: -16. 68 (*p* = 0.057); OARIS 0 vs 3: -18.00 (*p* = 0.041); OARSI 0 vs 4: -40.68 (*p* < 0.0001)). **D** The quantification of TUNEL positive cells in OA cartilage samples with different OA grades (Scale bar: 200 µm, Kruskal-Wallis test: F (4, 46) = 22.82, *p* < 0.0001, *N* > 8), (Dunns post-hoc, mean rank diff: OARSI 0 vs 1-2: -9.91 (*p* = 0.245); OARIS 0 vs 3: -15.13 (*p* = 0.045); OARSI 0 vs 4: -24.44 (*p* < 0.0001)). **E** A Ribo Green assay in SF showed that there is more RNA in the synovial fluid of OA patients with KL score 4 than in healthy controls OA patients with lower KL scores (one-way ANOVA: F (2, 20) = 4.302; *p* < 0.05, *N* > 7; KL 0-2: 3408 ng/ml, KL 3: 3265 ng/ml, KL 4: 4870 ng/ml). **F** IFNβ ELISA in SF of healthy samples and OA patients. (Kruskal-Wallis test: F (4, 38) = 8.39, p = 0.039, N > 6) (Dunn’s post-hoc, mean rank diff: OARSI 0-2 vs 3: -10.88 (p = 0.033); OARIS 0-2 vs 4: -5 (p = 0.65). **G** Semi-quantitative RT-PCR of TLR3 expression in human chondrocytes after treatment with siRNA to mediated TLR3 (upper panel). GAPDH is used as loading control. The graph below shows the poly(I:C)-induced IFNβ production in ELISA of siRNA treated chondrocytes compared to scrambled (scr) RNA-treated and untransfected chondrocytes (RM ANOVA: F (2, 6) = 11.73, *p* = 0.0084, *N* = 3). **H** IFNβ ELISA of human primary chondrocytes stimulated with poly(I:C) with and without previous treatment with TLR3 inhibitor CU CPT4a (RM ANOVA: F (1.59, 4.76) = 9.503, *p* = 0.02, *N* = 4).

To show the presence of TLR3 on chondrocytes, monolayer C28 chondrocytes were stained for TLR3 using immunohistochemistry. Using confocal microscopy, we found TLR3 to be present, as expected, on intracellular vesicular structures (Fig. 2A). To verify these results, we performed a western blot for TLR3 on primary human and murine chondrocyte lysates (Fig. 2B). The western blot confirms the presence of TLR3 in chondrocytes. To show a potential role of TLR3 in OA cartilage, we stained the cartilage of patients with different histological OA grades for TLR3 presence. The age distribution of the patients within the groups is comparable KL 0-2: 53 ± 21 years, KL 3: 64 ± 8 years and KL 4: 63 ± 8 years. We found a 30-fold increase in TLR3 positive chondrocytes in high OA-grade cartilage (OARSI4) compared to healthy controls (Fig. 2C; KL0 = 0.22 ± 0.24 (*N* = 15); KL1-2 = 0.67 ± 0.49 (*N* = 19); KL3 = 0.93 ± 1.2 (*N* = 17); KL4 = 6.02 ± 6.75 (*N* = 20); KL0 vs KL3 *p* < 0.05; KL0 vs KL4 *p* < 0.0001). OA samples were also analysed for programmed chondrocyte death using the TUNEL assay and again notably more TUNEL-positive cells could be found in OARSI3 and OARSI4 than in healthy cartilage (Fig. 2D; KL0 = 0.6 ± 1.02 (*N* = 12); KL1-2 = 4.18 ± 6.74 (*N* = 11); KL3 = 3.36 ± 2.39 (*N* = 8); KL4 = 9.78 ± 7.79 (*N* = 16); KL0 vs KL3 *p* < 0.05; KL0 vs KL4 *p* < 0.0001). Next, we investigated the presence of RNA concentration, as a potential ligand for TLR3, in the synovial fluid of OA patients (Age KL0-2: 58.4 ± 13.9 years; KL3 62.8 ± 11.8 years; KL4 64 ± 8.5 years). Interestingly, we found more RNA in OA than in healthy synovial fluid (Fig. 2E; KL0-2 = 3408 ± 1129 ng/ml (*N* = 8); KL3 = 3265 ± 573 ng/ml (*N* = 8); KL4 = 4870 ± 1612ng/ml (*N* = 7); KL0-2 vs KL4 *p* < 0.05). To investigate a possible activation of TLR3 signalling in OA, we quantified IFNβ in the synovial fluid of healthy controls and OA patients. We observed IFNβ to be noticeably increased in KL3 SF compared to KL0-2. However, the variance between the tested samples was quite high (Fig. 2F; KL0-2 = 41.95 ± 92.94 ng/ml (*N* = 11); KL3 = 1181 ± 1865ng/ml (*N* = 8), KL4 = 91.58 ± 80.49 ng/ml (*N* = 6); KL0-2 vs KL3 *p* < 0.05). To exclude an interference of signalling by other PRRs we knocked down TLR3 using siRNA in primary human chondrocytes. After stimulation with poly(I:C) these chondrocytes showed a noticeable decrease of IFNβ production compared to scr siRNA treated chondrocytes. Successful knockdown of TLR3 at mRNA level was proven by semiquantitative PCR (Fig. 2G; control = 0.035 ± 0.03 ng/ml (*N* = 3); scr siRNA = 0.269 ± 0.058 ng/ml (*N* = 3); TLR3 siRNA = 0.121 ± 0.08 ng/ml (*N* = 3); scr SiRNA vs TLR3 siRNA *p* < 0.05). To further validate this finding, we used CU CPT4a as a specific inhibitor for TLR3 and found a similar reduction of IFNβ secretion after stimulation with poly(I:C) in primary human chondrocytes (Fig. 2H; control = 1 ± 0 (*N* = 4); 0 μM = 1.27 ± 0.07 ng/ml (N = 4); 3.77 μM = 0.89 ± 0.15 ng/ml (*N* = 4); 7.54 μM = 0.8 ± 0.25 ng/ml (N = 4);control vs 0 μM p < 0.05).

### Inhibition of TLR3-dependent signalling protects mice from OA-like cartilage changes

**Fig. 3:**
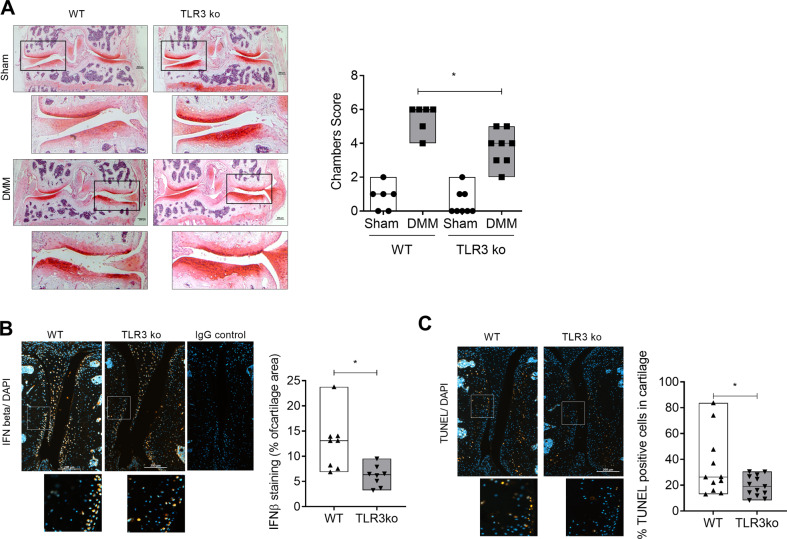
Inhibition of TLR3 dependent signalling protects mice from OA like cartilage changes. **A** Representative safranin-orange stained frontal knee joint sections of WT and TLR3^−/−^ mice after 8 weeks of DMM induction and the respective sham operated controls (Scale bar: 200 µm). The OARSI Score was used to quantify OA in the given graph (one-way ANOVA: F (3,24) = 51.60; *p* < 0.0001, WT *N* = 6, TLR3^−/−^ N = 8, Holm-Sidak post-hoc test DMM WT vs DMM TLR3 ko: 1.750, *p* = 0.002). **B** Representative pictures of IFNβ immunostaining in the medial knee joint compartment of DMM induced WT and TLR3^−/−^ mice. The quantification of IFNβ positive staining cartilage area is given in the graph (Scale bar: 200 µm, t-test: WT: 12.57 ± 1.92, TLR3^−/−^: 6.09 ± 0.73; *p* = 0.007, *N* > 6). **C** Representative TUNEL stainings of DMM induced medial compartment of WT and TLR3-/- frontal knee joint sections. Quantification of TUNEL positive cells in relation to number of DAPI positive nuclei TLR3^−/−^ mice also show fewer positive chondrocytes (Scale bar: 200 µm, t-test: WT: 34.87 ± 7.26, TLR3-/-: 19.64 ± 2.19; *p* = 0.042, *N* > 6).

To evaluate if the knockout of TLR3 might protect mice from degradative cartilage changes, knee OA was induced using the DMM model in 10-week-old wild type and TLR3^−/−^ mice. After eight weeks there was a difference between TLR3^−/−^ and wildtype OA knees (Fig. 3A; WT sham = 0.83 ± 0.75 (*N* = 6); WT DMM = 5.5 ± 0.04 (*N* = 6); TLR3^−/−^Sham = 0.5 ± 0.76 (*N* = 8); TLR3^−/−^ DMM = 3.75 ± 1.04 (*N* = 8); WT DMM vs TLR3^−/−^ DMM *p* < 0.05). IFNβ staining revealed that there was less IFNβ in the cartilage of TLR3^−/−^ mice eight weeks after surgery (Fig. 3B; WT DMM = 12.57 ± 5.43 (*N* = 8); TLR3^−/−^ DMM = 6.09 ± 2.07 (*N* = 8); *p* < 0.05). We also quantified apoptotic chondrocyte death in our murine knee joint sections to identify one possible source of TLR3 activation and subsequent IFNβ production. The TUNEL staining shows that there are fewer TUNEL positive chondrocytes in TLR3^−/−^ than in WT DMM cartilage (Fig. 3C; WT DMM = 34.87 ± 24.10 (*n* = 11, *N* = 6); TLR3^−/−^ DMM = 19.64 ± 7.89 (*n* = 13, *N* = 8); *p* < 0.05).

## Discussion

The most important finding of this study was that TLR3 contributes to degenerative changes in OA. In summary, the data shows that TLR3 is expressed in human and murine cartilage and that intra-articular release of ds nucleotides by apoptotic chondrocytes is one possible pathway causing TLR3 activation, subsequent IFNβ release and osteoarthritic changes within the joint.

TLR activation by DAMPs during OA has been demonstrated in recent studies [20–23]. A potential role of TLR3 in OA cartilage has been indicated by an up-regulation of gene expression and a description of functional polymorphisms in the promoter region that have been associated with a higher susceptibility for OA [12, 14]. Li and colleagues observed that in supernatants of damaged cartilage, TLR3 activation was the main stimulus for IL-33, MMP-1 and MMP-3 expression in vitro [16]. Furthermore, increased IFNβ expression after TLR3 activation has already been shown in RA synovial tissue and fibroblasts [24]. However, while mounting evidence of TLR3 presence during the pathogenesis of OA has been presented, the underlying mechanisms of TLR3-dependent signalling contributing to the disease progression remain elusive. To shed further light on the role of TLR3 in cartilage degradation during OA our study confirms the presence of TLR3 on the RNA and protein level in cartilage. Cartilage samples of patients with OA showed significantly more TLR3 positive cells than healthy controls, which proves the clinical relevance of TLR3 in cartilage.

Chondrocyte death has been identified in the early stages of OA [22, 25]. Programmed or necrotic cell death causes the release of both dsDNA and dsRNA, making TLR3 an endogenous sensor of tissue necrosis [16, 17, 26]. Contrary to previous reports, recent literature suggests that chondrocytes are the key player in inflammatory changes in OA. Zhang *et al*. demonstrated that the induced death of superficial chondrocytes protects from degenerative cartilage changes [18]. Similarly, the removal of senescent chondrocytes in a post-traumatic OA mouse model attenuates disease progression [19]. However, the mechanisms driving these inflammatory changes are not yet clear. The data of this study shows a significant increase of dsRNA in the supernatant of human OA synovial fluid in vivo and in vitro after staurosporine stimulation in comparable concentrations. Chondrocytes are able to take up these potential TLR ligands even through the dense peri-cellular matrix to the endosomal location of TLR3 [27]. The confocal imaging in this study indicates that nucleic acid is taken up in cartilage tissue as well as in monolayer chondrocytes. Furthermore, biomechanical studies suggest that deterioration of the collagen-proteoglycan network such as in OA increases cartilage permeability which again might ease the access of inflammatory ligands to the chondrocytes [17, 28–30]. Thus, ds nucleotides are a possible mediator between chondrocyte death and induction of inflammatory changes.

Our results show that TLR3 is up-regulated in OA, which induces a pro-inflammatory cell response [31]. TLR3 downstream signal transduction results in IFNβ production [16, 32]. However, the effect of IFNβ on inflammation in OA continues to be a topic of basic and clinical research [33–35]. Furthermore, other PRRs such as the RIG-1, MDA-5 and TLR-7, -8, -9 also recognize nucleic acids and might also contribute to the described inflammatory changes [13]. This might be the reason why TLR3 knockdown chondrocytes did not show a complete suppression of IFNβ in this study. However, specific TLR3 inhibition by CU CPT 4a was highly effective. Future research is required to expand the knowledge about the role of other nucleotide-binding endogenous TLRs and PRRs in OA [36, 37].

So far, there are two in vivo studies investigating the effect of TLR3 in mediating joint degeneration. Zare *et al*. used intra-articular poly(I:C) injections and observed no significant difference in synovial inflammation between TLR3^−/−^ and WT mice. However, they sacrificed the mice after three days, investigating only the initial inflammatory reaction [15]. Li et al. demonstrated that inflammatory changes caused by intra-articular Poly(I:C) injections were significantly dampened by an IL-33-neutralizing antibody [16]. Both models, however, did not investigate the long-term effects of joint destabilization in an OA mouse model. By destabilizing the medial meniscus we chose a well-established OA model that mimics post-traumatic OA rather than chemically induced OA [38]. We found that the knockout of TLR3 significantly reduced the OA-related cartilage degradation in the animals and slowed down OA progression by up to eight weeks. Future research needs to evaluate the systemic and synovial inflammatory effect of TLR3 on OA progression and long-term effects. Furthermore, the data of this study does not clarify to what extent TLR3 activation causes programmed chondrocyte death and whether joint injury causes cell death, then inducing TLR3-based inflammation [39]. While human OA cartilage samples show an increase of both TUNEL and TLR3 positive cells, TLR^−/−^ mice showed significantly fewer TUNEL positive chondrocytes. This indicates that TLR3-induced programmed cell death plays a secondary role in the pathology of OA [36]. However, literature shows clear evidence of inflammation-based TLR3-mediated cartilage degeneration, which leaves this question unanswered [16].

Recent OA research has been experiencing a paradigm shift in that it shows that sterile inflammation caused by DAMPs contributes to the disease’s progression [36]. Our work indicates that joint trauma induces chondrocyte death and the subsequent release of ds nucleotides. As suggested by other authors, chondrocytes seem to be key drivers of degenerative cartilage changes and are able to take up nucleotides [18, 19]. Our in vivo experiments underline these in vitro findings and show that TLR3 knockout protects mice from OA-like cartilage degradation. Thus the inhibition of TLR3 signalling could be a possible therapeutic target for osteoarthritis.

## Supplementary information

Original Data File

Supplemental Material

Reproducibility checklist

## Data Availability

Data is available upon reasonable request.
