# Supplementary figures and images for: Toll-like receptor 3 activation promotes joint degeneration in osteoarthritis
Source: Cell Death Dis. 2022 Mar 11;13(3):224. doi: 10.1038/s41419-022-04680-5 (PMC8917184; doi:10.1038/s41419-022-04680-5)

PCR\_hTLR3789\_GAPDH

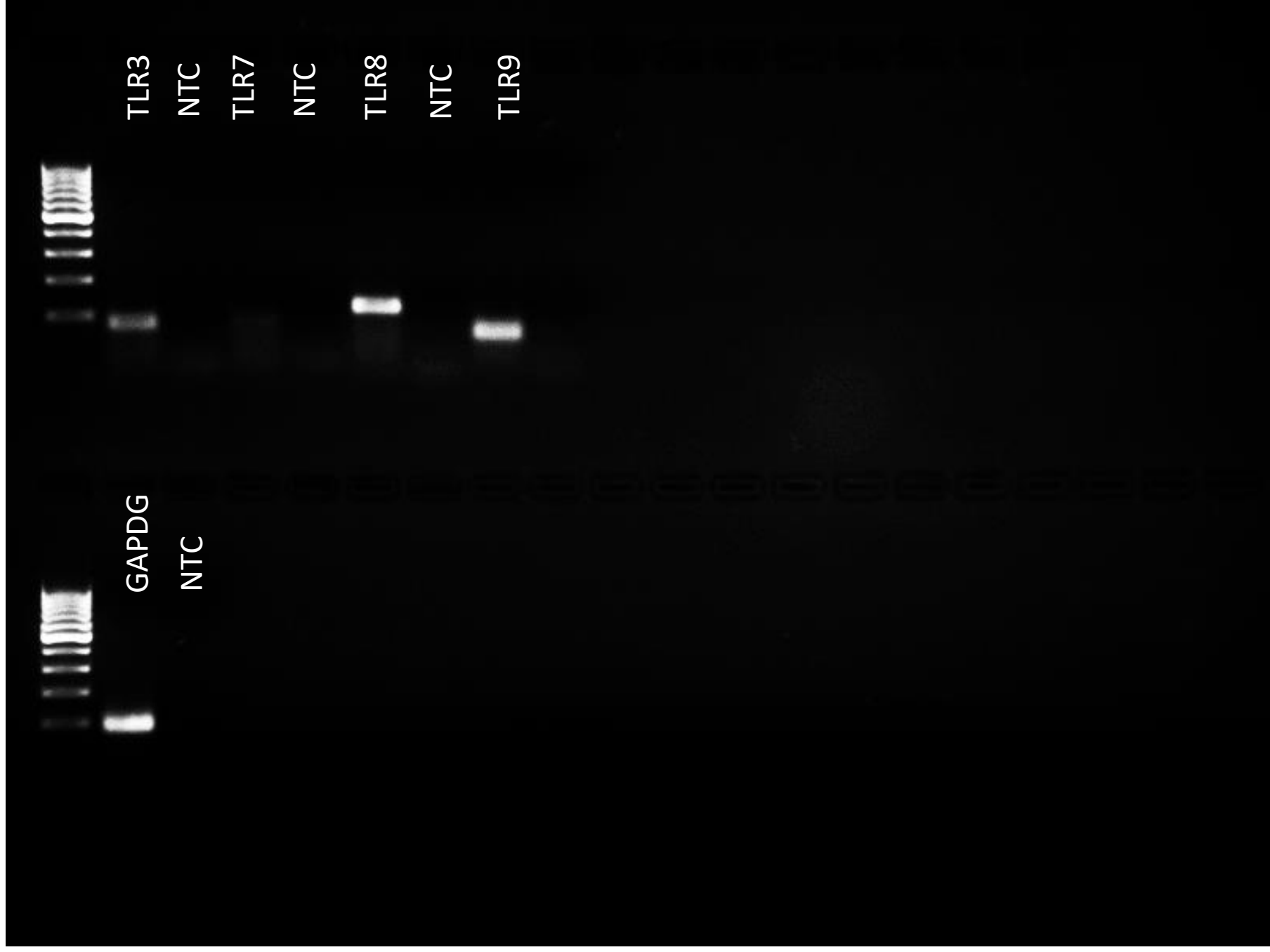

PCR\_mTLR3789\_GAPDH

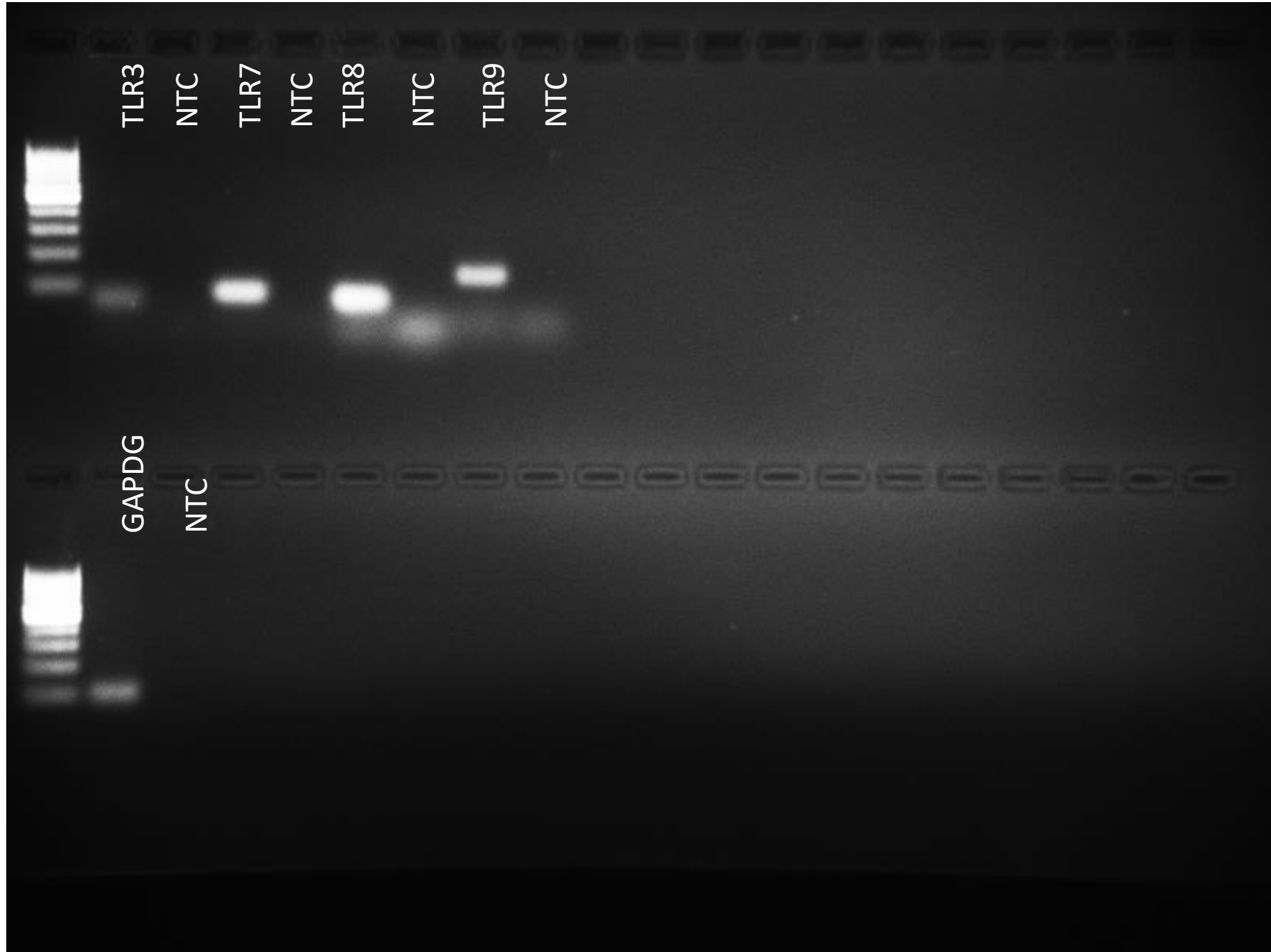

Fig. 2B

TLR3 Blot mouse human

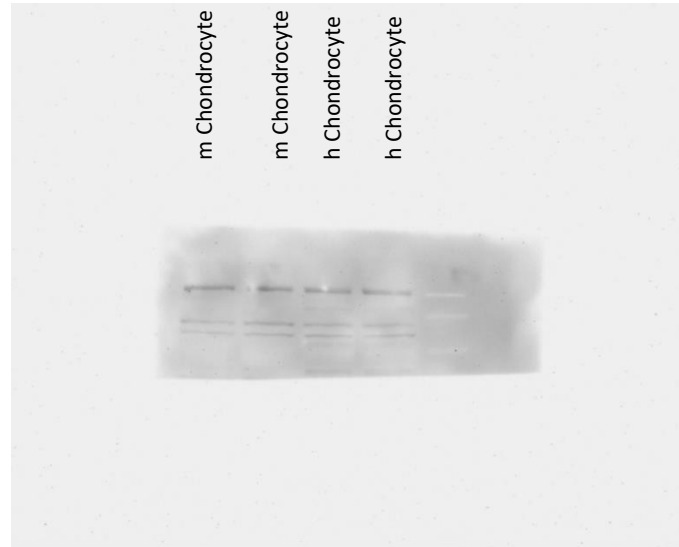

GapDH of TLR3 Blot mouse human

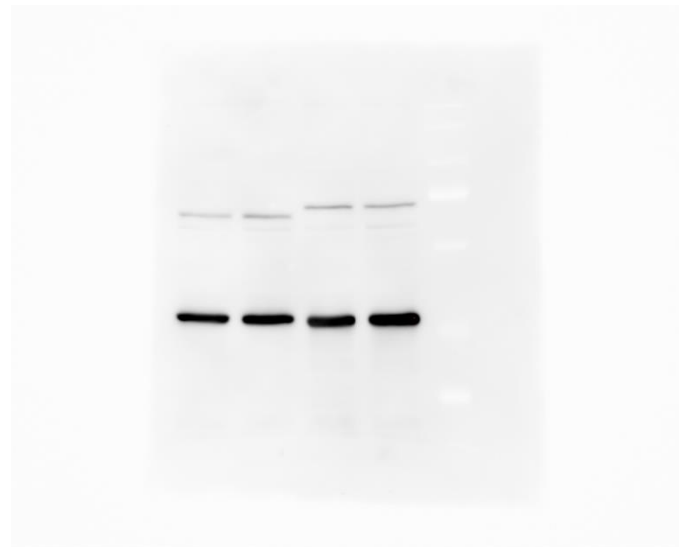

Fig. 2G

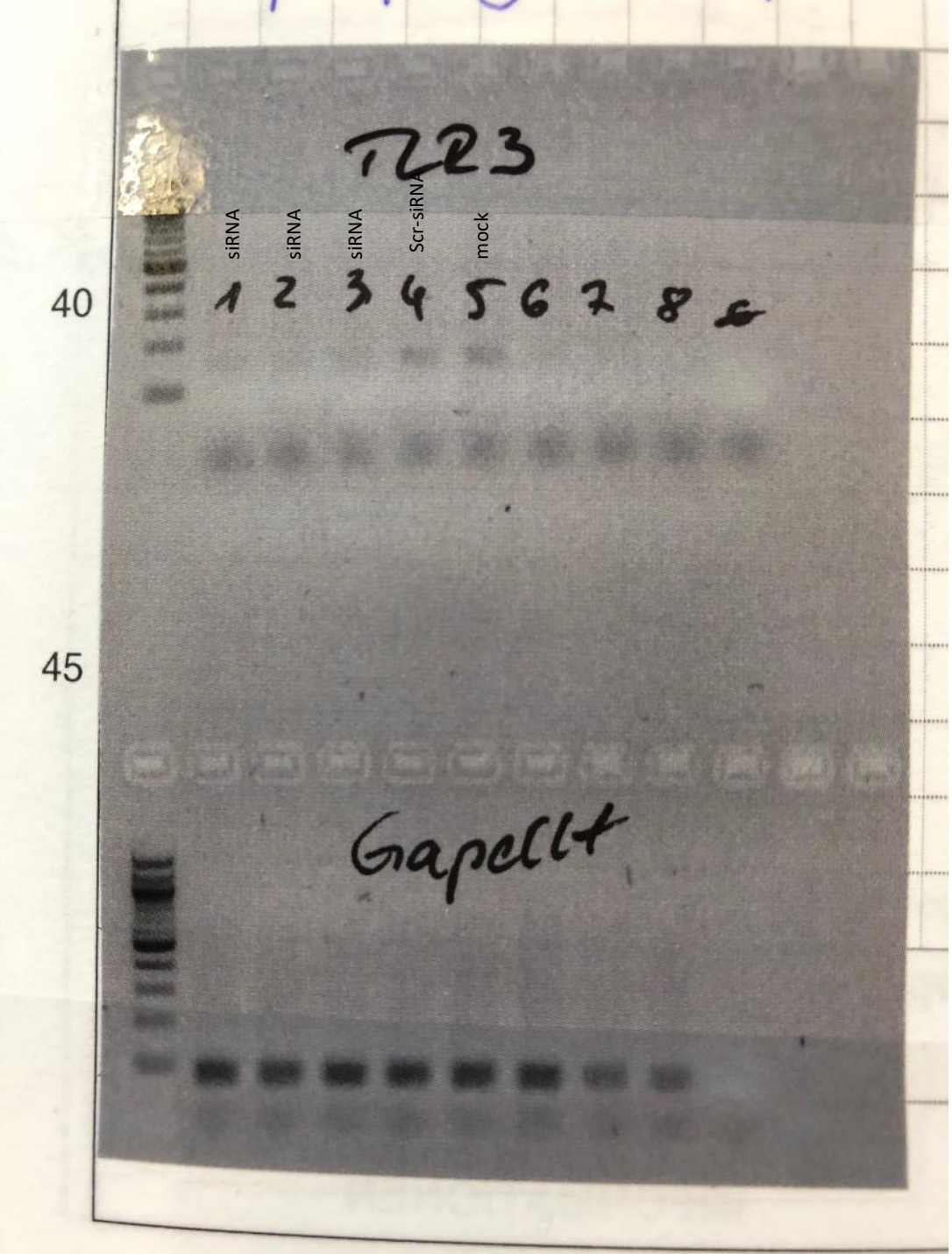

Supplement: Supplementary file 1 — Original Data File [file 41419_2022_4680_MOESM1_ESM.pdf]
