# Supplementary material for: Toll-like receptor 3 activation promotes joint degeneration in osteoarthritis
Source: Cell Death Dis. 2022 Mar 11;13(3):224. doi: 10.1038/s41419-022-04680-5 (PMC8917184; doi:10.1038/s41419-022-04680-5)
Supplement: Supplementary file 2 — Supplemental Material [file 41419_2022_4680_MOESM2_ESM.docx]

**Suppl. Table:**

*Suppl. Table 1: Primers*

| Primer | forward (5' to 3') | reverse (5' to 3') |
| --- | --- | --- |
| hTLR3 | GCC ACA CAC TTC CCT GAT GA | CAT GAT TCT GTT GGA TGA CTG CT |
| hTLR7 | TCT TGG CAC CTC TCA TGC TC | TGT CCA CAT TGG AAA CAC CAT TT |
| hTLR8 | AAA CAT GGT TCT CTT GAC ACT TCA G | GGC TGC AGG AGC TAT TTT GC |
| hTLR9 | AGA TGT AAG CGC CAA CCC TCT G | TCC AGC AGG AAG TCC ATA AAG GC |
| hGAPDH | CCC ACT CCT CCA CCT TTG AC | AGC CAA ATT CGT TGT CAT ACC AG |
| hMRPS7 | GAG GCC GTT TCT ACC AGG TC | TCC TGG AGA CTC TAC CAC CA |
| mTLR3 | AGG CAG AAG TTT GGT CCA GAC C | TGG GCC TGC TTG AGT AAG AAA GG |
| mTLR7 | AAG AAA GAT GTC CTT GGC TCC C | TCA AGA GGT CTG GTG GAG GA |
| mTLR8 | ACT CAC AGT ACC TGA GGC TTC G | ATT GTT GGG CCA CTG GAG GAT G |
| mTLR9 | TCC TCC ATC TCC CAA CAT GGT TC | TTC AGC TCA CAG GGT AGG AAG G |
| mGAPDH | AGC AAG GAC ACT GAG CAA GAG AGG | GGG TCT GGG ATG GAA ATT GTG AGG |
